# Abnormal Network Homogeneity in the Right Superior Medial Frontal Gyrus in Cervical Dystonia

**DOI:** 10.3389/fneur.2021.729068

**Published:** 2021-11-05

**Authors:** Shubao Wei, Xiuqiong Chen, Yousheng Xiao, Wenyan Jiang, Qiong Yin, Chunhui Lu, Lu Yang, Jing Wei, Yang Liu, Wenmei Li, Jingqun Tang, Wenbin Guo, Shuguang Luo

**Affiliations:** ^1^Department of Rehabilitation Medicine, Jiangbin Hospital of Guangxi Zhuang Autonomous Region, Nanning, China; ^2^Department of Neurology, The First Affiliated Hospital of Guangxi Medical University, Nanning, China; ^3^Department of Radiology, The First Affiliated Hospital of Guangxi Medical University, Nanning, China; ^4^National Clinical Research Center for Mental Disorders, Department of Psychiatry, The Second Xiangya Hospital of Central South University, Changsha, China

**Keywords:** cervical dystonia, resting-state functional magnetic resonance, network homogeneity, default mode network, superior medial frontal gyrus

## Abstract

**Background:** Increasing evidence from modern neuroimaging has confirmed that cervical dystonia (CD) is caused by network abnormalities. Specific brain networks are known to be crucial in patients suffering from CD. However, changes in network homogeneity (NH) in CD patients have not been characterized. Therefore, the purpose of this study was to investigate the NH of patients with CD.

**Methods:** An automated NH method was used to analyze resting-state functional magnetic resonance (fMRI) data from 19 patients with CD and 21 gender- and age-matched healthy controls (HC). Correlation analysis were conducted between NH, illness duration and symptom severity measured by the Tsui scale.

**Results:** Compared with the HC group, CD patients showed a lower NH in the right superior medial frontal gyrus. No significant correlations were found between abnormal NH values and illness duration or symptom severity.

**Conclusion:** Our findings suggest the existence of abnormal NH in the default mode network (DMN) of CD patients, and thereby highlight the importance of the DMN in the pathophysiology of CD.

## Introduction

Cervical dystonia (CD) is a disorder of the nervous system characterized by involuntary sustained contractions of the cervical musculature, causing an abnormal and involuntary rotation or tilt of the head in specific directions (1). The head may typically turn resulting in torticollis, laterocollis, anterocollis, or retrocollis (2). CD is the most common type of focal dystonia, and the first symptoms are frequently exhibited between 40 and 60 years of age. The disorder is often accompanied by head tremors and chronic neck pain (3). Abnormal posture can cause pain and lead to significant disability in daily living activities, such as reading and driving (4, 5), which can lead to unemployment (6, 7). Indeed, there has been reported a substantial negative influence of CD on employment, with CD-related pain as a particularly important factor (8). Moreover, CD patients sustain significant psychosocial disability and a decline in their quality of life. Therefore, it is important to diagnose patients with CD and offer these patients an effective therapy, however, the pathological mechanism of CD is still poorly understood.

It is widely accepted that deficient motor control in dystonia is related to basal ganglia dysfunction, ultimately resulting in a systems-level loss of inhibitory function (9–12). Recently, emerging evidence suggests the probability of widespread abnormalities in brain networks in which the basal ganglia are important nodes. This introduced the hypothesis that CD can be attributed to the dysfunction of specific brain networks, although their precise role in CD is currently uncertain.

Through the developments of neuroimaging techniques, it is now possible to investigate details of structural changes and neuronal activity in the brain that are potentially involved in the pathophysiology of CD. For example, several structural neuroimaging studies have reported micro-structural changes in specific brain regions of CD patients, including the basal ganglia, thalamus, cerebellum, motor cortex, and supplementary motor cortices (13–21). Functional magnetic resonance imaging (fMRI) studies provide the most consistent results (22, 23). Furthermore, in task-free fMRI research, CD patients were shown to have altered functional brain connectivity in a couple of resting-state networks as compared with those of healthy controls (22). Thus, cervical dystonia is suggested to be a disorder caused by the dysfunction of multiple neural networks. Recently, we have studied regional homogeneity (ReHo) changes in CD, and confirmed that abnormal ReHo existed in brain regions of the “pain matrix”, salience network (the right insula and bilateral middle cingulate gyrus), motor network (the right precentral gyrus), cerebellum and medial prefrontal cortex (MPFC) (24). However, it remains unclear if the network homogeneity (NH) is affected in CD patients.

Over recent years, a methods to analyze resting-state fMRI data have provided a new way to study the previously neglected field of intrinsic network organization (25). The method named NH, which is an unbiased assessment of the homogeneity of a neural network, was first used to study the default mode network (DMN) of attention-deficit/hyperactivity disorder by Uddin et al. (25). NH is a voxel-wise that examines the correlation of a voxel with all other voxels belonging to a specific network of interest. The NH existed its advantages that have been detail stated in our previous studies (26, 27). Homogeneity is defined as the average correlation of the time series of any given voxel with the time series of all other voxels within the network (25). So far, NH has been investigated in studies of individuals with somatization disorder (26), major depressive disorder (28), schizophrenia (27) and their unaffected counterparts (29).

In this study, we used the NH method to study patients with CD. Previously, we have used Global-brain functional connectivity (GFC), voxel-mirrored homotopic connectivity (VMHC) and ReHo to analyze fMRI data of patients with CD. Results showed that the GFC and the VMHC in the supplementary motor area (SMA) were significantly decreased (30, 31). Furthermore, a negative correlation was found between the VMHC values in the SMA with the severity of dystonia (31). Additionally, CD patients showed increased ReHo in the right cerebellum crus I and decreased ReHo in the right superior MPFC. The right precentral gyrus, right insula, and bilateral middle cingulated gyrus also showed increased ReHo values. A significantly positive correlation was observed between ReHo in the right cerebellum crus I and symptom severity (24). Therefore, CD patients had alterations in brain networks of the “pain matrix”, salience network, motor network and DMN. Given the NH method was restricted to reveal the neuro-activities of DMN, thus we expected the NH method making new contributions by exploring NH in the DMN to understanding the pathological mechanism of CD. Based on previous findings, we hypothesize that CD patients would show abnormal NH, particularly the DMN, and that may further positively correlated with clinical data such as illness duration and symptom severity.

## Materials and Methods

### Subjects

This study, which was approved by the ethics committee of the First Affiliated Hospital, Guangxi Medical University, China, was performed in an outpatient setting in the Department of Neurology of The First Affiliated Hospital. Patients were diagnosed based on the 2011 EFNS (European Federation of Neurological Societies) guideline on the diagnosis and treatment of primary dystonias. Patients who matched the following criteria were included in the study: (1) idiopathic cervical dystonia; (2) no history of botulinum toxin treatment, related drug therapy, or operation in the previous 3 months; (3) no history of serious physical or neuropsychiatric disorders; and (4) right-handedness.

Healthy controls (HC) were simultaneously recruited from the community. They were group-matched in gender, age, and were all right-handed. The exclusion criteria for HC individuals were as follows: (1) diagnosis of secondary spasmodic torticollis; (2) any history of severe physical or neuropsychiatric disorders; and (3) any family history of psychiatric or neurological diseases in their first-degree relatives. Only subjects who had no contraindications for MRI or exhibited no abnormalities in conventional MRI scans were included.

The Tsui scale (32) was used to measure the symptom severity of CD of all patients. Every subject was given information relating to study procedures and provided written informed consent.

### Image Acquisition

Resting-state image acquisition was performed on a Siemens 3.0 T scanner (Erlangen, Germany). All subjects underwent scans with foam padding and earplugs to minimize head movement and reduce scanner noise. Every participant was asked to lie still and relax, eyes closed, and remain awake but avoid thinking of anything in particular. Subsequently, we confirmed that each subject had not fallen asleep during the session. For each subject, the following parameters were used for echo-planar imaging sequence acquisition: repetition time/echo time (TR/TE) = 2,000/30 ms, slice thickness = 4 mm, flip angle = 90°, gap = 0.4 mm, matrix = 64 × 64, FOV = 24 × 24cm, number of volumes = 250.

### Data Pre-processing

Data pre-processing was performed using Matlab (33) (Mathworks) data processing assistant for resting-state fMRI (DPARSF) (34). The primary process included: slice timing, realignment, spatial normalization, registration to the MNI template, and temporal bandpass filtering. Details of preprocessing are presented in our previous study (26). We excluded the participants who had more than 2° of angular rotation in each axis and 2 mm of translation in the x-, y-, or z-direction.

### NH Analysis

After data pre-processing, we performed NH analysis on Matlab (33) (Mathworks). The equation to calculate the NH of CD patients and HCs has been previously described by Uddin et al. (25). Briefly, the correlation coefficients of each voxel were computed against all other voxels within the DMN mask and averaged to generate the NH maps. Subsequently, the NH maps were smoothed by a Gaussian kernel of 8 mm with full-width at half-maximum and used in further analysis.

### Statistical Analysis

Demographic and clinical information, such as sex and age were recorded. The Chi-square test was used to compare qualitative variables, whereas the two-sample *t*-test was used to compare quantitative variables (*p* < 0.05). The NH analysis were completed using REST with a two-sample *t*-test. Multiple comparisons were conducted using a Gaussian random field (GRF) correction (voxel significance: *p* < 0.001, cluster significance: *p* < 0.005), and the level of significance was set at the corrected *p* < 0.05.

Moreover, the mean NH values of brain regions that were defined as regions of interest (ROIs) that showed abnormal NH were extracted. Subsequently, further correlation analysis between these NH values and illness duration and Tsui scores in the patient group (*p* < 0.05) were performed. Correlation analysis of mean NH values of clusters and patient age were performed using Pearson's correlations, and correlation analysis of illness duration and Tsui total score were performed using Spearman's correlations.

### Reproducibility

Given the small sample size, we used split-half and leave-one-out validations to assess the reproducibility of the present results. First, we randomly selected 10 CD patients and 11 healthy controls from the two groups and analyzed using the same statistical tests as those employed for the total number of patients. In parallel, group comparisons may significantly lose statistical power in the split-half analysis. Thus, we also performed leave-one-out validations of the reproducibility and robustness of these results without reduced statistical power. Specifically, we excluded one CD patient from the group and performed the same group comparisons based on the permutated sample (e.g., 18 CD vs. 21 healthy controls). Consequently, a total of 19 two-sample *t*-test images were generated. Based on these images, numerous tests for each voxel revealed significant group differences across all 19 two-sample *t*-tests; these differences were calculated as the reproducibility of NH between CD patients and HCs.

## Results

### Subjects

There were no subject excluded due to any contraindications for MRI or shown changes under conventional MRI scans. No participants were excluded due to falling asleep during image acquisition. Two patients were excluded due to excessive head movement. Therefore, 19 patients and 21 HCs were included in this study. As listed in Table 1, there were no significant differences between the patient and control groups in gender ratio (*t*-tests, *p* = 0.220) or age (Chi-square test, *p* = 0.759). There were no difference in head motion of the residual patients and HCs. The detail results were listed in Table 2. Additionally, in the CD group, 12 patients were bilaterally affected, while the remaining patients were affected on the left side only. A total of 17 patients complained of painful neck muscles, and 18 of 19 CD patients reported sensory tricks. Sensory tricks are known as an abnormal posture and involuntary movements of the head and neck that can be temporarily improved by specific behaviors, including light touching the lower part of the cheek, jaw and posterior neck, leaning against the wall, carrying a backpack or keeping something in the mouth.

**Table 1:** Demographics and clinical characteristics of the patients and the controls.

**Variables** **(mean ± standard deviation)**	**Patients**	**Controls**	***p-*value**
Gender (female/male)	10/9	15/6	0.220^a^
Age, years	38.74 ± 10.71	39.62 ± 6.62	0.759^b^
Illness duration, months	24.29 ± 31.26		
Tsui	16.32 ± 4.45		

*Tsui, Tsui scale*.

a *The p-value for gender distribution in the two groups was obtained by chi-square test*.

b *The p-values were obtained by two sample t-tests*.

**Table 2:** Difference in head motion of the patients and the controls.

**Testing statistic**	**Translation**			**Rotation**		
	**x**	**y**	**z**	**x**	**y**	**z**
*T*	−0.943	−0.781	−0.033	−0.378	−0.845	−1.148
*P*	0.352	0.439	0.974	0.707	0.403	0.258

*The p values were obtained by two sample t-tests. x, y, z, coordinates of the MNI space. x, y, z represented of translation in the x-, y-, or z-direction and angular rotation in each axis*.

### NH: Between-Group Comparison

**Figure 1:**
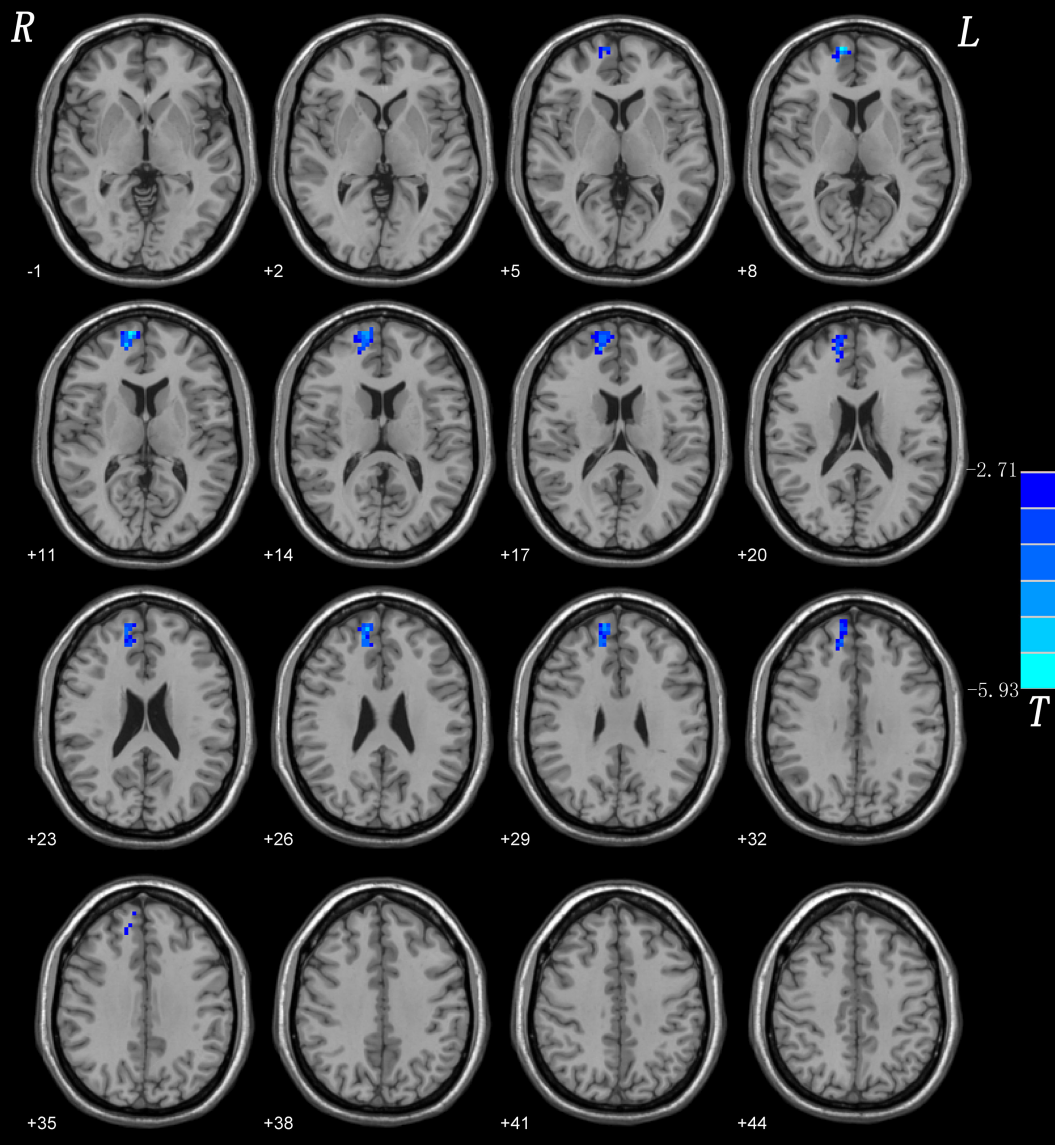
NH differences between patients with CD and controls. Red and blue denote higher and lower NH respectively and the color bars represent the t values from two-sample *t*-test of the group analysis. NH, network homogeneity; CD, cervical dystonia.

Compared with HCs, CD patients showed a lower NH in the right superior medial frontal gyrus. None of the brain regions showed a significantly increased NH in CD patients compared with those of HCs. Group comparisons of NH values are shown in (Figure 1; Table 3).

**Table 3:** Brain regions with significant NH differences in the patients.

**Brain regions**	**Peak (MNI)**			**Number of voxels**	***T-*value**
	**x**	**y**	**z**		
*Patients < Controls*					
Right superior medial frontal gyrus	12	63	12	90	−5.9244

*x, y, z, coordinates of primary peak locations in the MNI space; T, statistical value of peak voxel showing NH differences between the patients with CD and the controls; CD, cervical dystonia; NH, network homogeneity; MNI, Montreal neurological Institute*.

### Reproducibility

Considering the small sample size of our study, we used split-half and leave-one-out validations to assess the reproducibility of our results.

#### Split-Half

**Figure 2:**
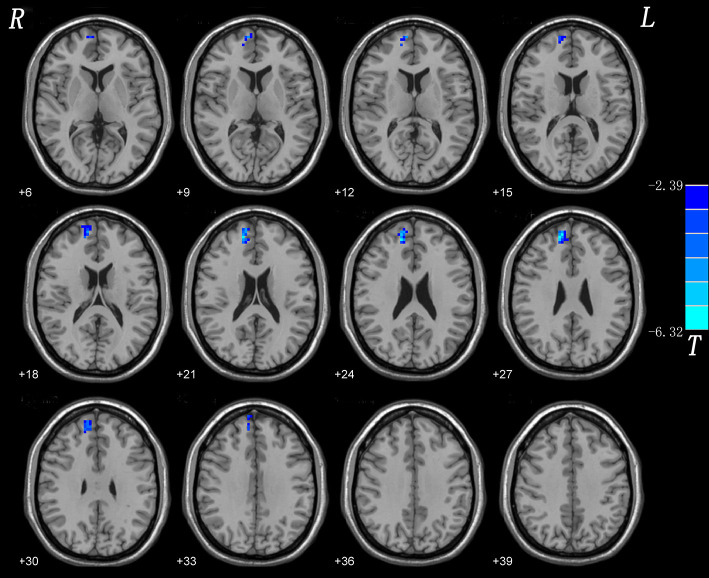
Split-half validation. Comparisons of whole-brain NH were performed between 10 CD patients and 11 healthy controls. Blue denote lower NH respectively and the color bars represent the t value from two-sample *t*-test of the group analysis. NH, network homogeneity; CD, cervical dystonia.

The results of split-half replicated those of the full sample (Figure 2).

#### Leave-One-Out Validation

**Figure 3:**
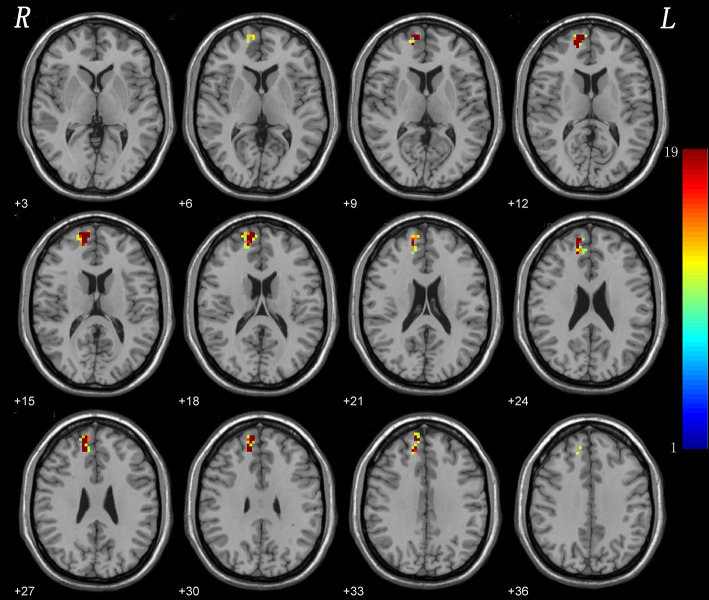
Leave-one-out validation. The group comparisons based upon the permutated samples (i.e., 18 CD vs. 21 healthy controls) for total 19 times. For each voxel, the color indicates number of tests where this voxel exhibited significant group differences across the total 19 tests (i.e., the reproducibility).

The results indicated a highly reproducible pattern of NH alteration across these tests, as well as in the overall analysis (Figure 3).

### Correlations Between NH and Clinical Variables

The abnormal NH in the right superior medial frontal gyrus of CD patients was defined as the ROI, from which mean NH values were extracted. Linear correlations were calculated between these NH values and the illness duration or Tsui score in the patient group. No significant correlation was detected between these NH values and illness duration or Tsui score.

### ROC Results

**Figure 4:**
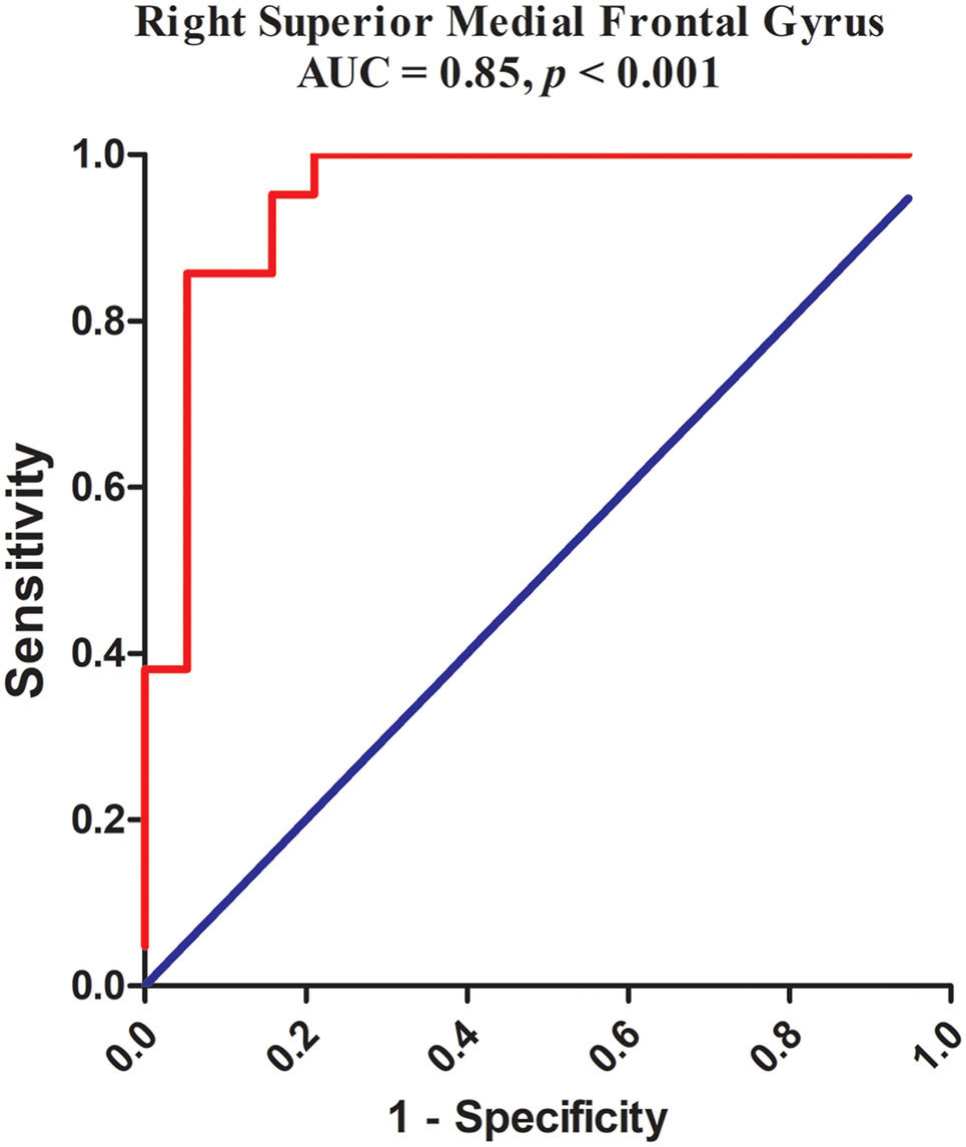
Receiver operating characteristic (ROC) curves using the mean NH in the right superior medial frontal gyrus to separate the patients from healthy controls. NH, network homogeneity.

The abnormality found for NH values in the right superior medial frontal gyrus of CD patients was further validated by the ROC method. The results supported that the NH values in the right superior medial frontal gyrus could be applied to differentiate patients from controls with optimal specificity (85.71%, 18/21) and sensitivity (94.74%, 18/19) (Figure 4; Table 4).

**Table 4:** ROC analysis for differentiating patients from healthy controls.

**Brain regions**	**Area under the curve**	**Cut-off point**	**Sensitivity**	**Specificity**
Right medial superior frontal gyrus	0.950	0.80	94.74%, 18/19	85.71%, 18/21

*ROC, receiver operating characteristic; NH, network homogeneity*.

## Discussion

Previous fMRI studies in CD patients were mostly task-based, which have limitations compared with resting-state fMRI. First, the introduction of tasks is likely to cause activation in brain regions associated with the completion of tasks and mask abnormal spontaneous activities in regions associated with the pathogenesis of the disease. In addition, it is difficult to choose tasks related to CD symptoms based on MRI scans. Thus, we used the resting-state fMRI method to study CD patients. NH analysis is a new method to evaluate the NH of a distributed and meaningful network based on the level of a voxel-wise comparison. This study used the NH method to analyze the fMRI data of CD patients and HCs. Compared with the HC group, CD patients showed lower NH values in the right superior medial frontal gyrus. No brain region had higher NH values in the CD group compared with those of the HC group. Furthermore, no significant correlations were found between abnormal NH values and age, illness duration, or symptom severity.

The right superior medial frontal gyrus is a primary region of the SMA (35, 36). The SMA, is an important node of the cortical-basal ganglia loop, as well as a complement to the primary motor area and is involved in voluntary movement and advanced motor activities, such as movement and language initiation. This área couples the afferent input and efferent output of the motor cortex area and integrates complicated movement sequences with memory organization (36). Additionally, it is linked to the primary motor cortex, premotor area, and anterior cingulate area. Therefore, decreased NH values of CD patients in the right superior medial frontal gyrus may be implicated in the involuntary spasms of focal muscles. In the aforementioned studies, the SMA was found to have a decreased function when analyzed using different analytical methods (i.e., VMHC and GFC) (30, 31). These findings strongly suggest that SMA dysfunction plays a key role in CD occurrence.

Of the 19 CD patients we included, 17 patients showed “sensory tricks”. Neurophysiological evidence suggests that sensory tricks work by reducing abnormal facilitation. Moreover, this is associated with established dystonia pathogenesis, leading to the hypothesis that sensory tricks reduce abnormally increased facilitation-to-inhibition ratios in brains with dystonia (37). Naumann et al. found that CD patients had a decreased activation of the contralateral SMA, primary sensorimotor cortex, ipsilateral parietal regions while the bilateral occipital lobe was overactivated upon “sensory tricks” stimulation (38). On the basis of these observations, we postulate that CD patients exhibit disturbed SMA function, leading to abnormal initiation, planning of movement, and advanced motor functions, which may be involved in the mechanism of sensory tricks. Thus, we hypothesize that the right superior medial frontal gyrus affects the function of the SMA thereby contributing to the onset of CD.

The right superior medial frontal gyrus was suggested as a key node of the DMN (39). Besides, a neuroimaging study that investigated several subregions of the human superior frontal gyrus (SFG) showed a strong functional connectivity with the anterior cingulate cortex of the DMN (40). The DMN performs functional connectivity enhancement and exhibits a high level of neural activity in the resting state but it is inactivated when performing targeted tasks or behaviors. Its main function is to maintain self-referential activity, monitoring the self and surrounding environment (41), and environmental positioning (42). The DMN is known to exhibit abnormal neuro-activity in several neurological and neuropsychiatric disorders, such as Alzheimer's, Parkinson's, epilepsy, schizophrenia (27, 43, 44), and major depressive disorder (28). In another study, we observed abnormal regional activity of this network (the right cerebellum crus I and superior medial prefrontal cortex) (24). Lower NH values of the right superior medial frontal gyrus in CD patients might therefore influence the function of this region and result in loss of top-down regulation. This might be a basal pathological change associated with self-referential activity, self and environmental monitoring, and environmental positioning, consequently disturbing posture control. In another word, lower NH in the right superior medial frontal gyrus contributed to disturb the DMN function on posture controlling or rather contraction of the cervical musculature involuntary and sustained in CD patients. Thus, the right superior medial frontal gyrus was an evidence of abnormal function of DMN in patients with CD. On this basis, we speculate that the DMN may also be involved in the occurrence of CD therefore it could be expected as a potential biomarker for further study.

We observed asymmetric activity patterns in CD that were primarily involved in the right-hemispheric dystonia-related connectivity pattern. It is important to note that the asymmetric activity patterns were not an accidental finding, as we have noted in other studies in patients with CD (24). This phenomenon has been previously reported, for instance, during finger movements (45) and in the resting state (22). A possible explanation for the laterality of the right hemisphere may be due to its dominance in position control; i.e., the right hemisphere determines response modifications so that it is dominant for position control (46). However, how this pattern relates to the pathogenesis of CD remains to be further studied.

Strikingly, no significant correlations were found between abnormal NH brain regions and CD clinical data such as illness duration and severity of symptoms. This suggests a corollary that the abnormality of this region is an inherent abnormality of dystonia. The most likely reason is that abnormalities in the cortex and not only the basal ganglia are a cause for this disorder. Thus, we suggest that abnormalities in the right superior medial frontal gyrus are intrinsic to CD. In addition, as presented in Table 3, the sensitivity and specificity of the ROC analysis in the right superior medial frontal gyrus were 94.74 and 85.71%, and the area under the curve of the right superior medial frontal gyrus was >0.7, an acceptable accuracy for established diagnostic indicators. Hence, we suggest that decreased NH in the right superior medial frontal gyrus might be utilized as a potential biomarker to diagnose CD patients.

There are several limitations to our study. First, the sample size was small. Second, dystonic posturing was minimal in the supine position or absent during scanning in all patients. To confirm whether this was a specific sensory trick is challenging. Therefore, the influence of a sensory trick on the analysis could not be fully eliminated. Moreover, physiological noises including respiratory and heart rhythms could not be eliminated despite the use of a low sampling rate (TR = 2s). Lastly, given the small sample size, patients were not subdivided into different groups according to head rotation. Hence, research using a larger sample size is required to expand our findings.

## Conclusions

Our findings suggest that abnormal NH of the DMN is a key feature of CD patients, which further highlights the importance of the DMN in the pathology of CD. The DMN may be exploited as a potential biomarker to diagnose CD patients.

## Data Availability Statement

SL had access to all the data in the study and had final responsibility for decision to submit for publication. The data will be available upon request to SL, luoshuguang@stu.gxmu.edu.cn.

## Ethics Statement

The studies involving human participants were reviewed and approved by the Ethics Committee of The First Affiliated Hospital, Guangxi Medical University. All participants were given information regarding study procedures and subsequently provided written informed consent to participate in this study.

## Author Contributions

SL and WG: conceived and designed the study. SW, XC, YX, WJ, QY, CL, LY, JW, YL, WL, and JT: collected the original imaging data. WG: managed and analyzed the imaging data. SW and XC: wrote the first draft of the manuscript. All authors contributed to the article and approved the submitted version.

## Funding

This study was supported by grants from the National Key R&D Program of China (Grant No. 2016YFC1307100), National Natural Science Foundation of China (Grant No. 81771447), Natural Science Foundation of Hunan (Grant No. 2020JJ4784), Guangxi Appropriate Technology for Medical and Health Research and Development Project (Grant No. S2020028), and Guangxi Appropriate Technology for Medical and Health Research and Development Project (Grant No. S2019002).

## Conflict of Interest

The authors declare that the research was conducted in the absence of any commercial or financial relationships that could be construed as a potential conflict of interest.

## Publisher's Note

All claims expressed in this article are solely those of the authors and do not necessarily represent those of their affiliated organizations, or those of the publisher, the editors and the reviewers. Any product that may be evaluated in this article, or claim that may be made by its manufacturer, is not guaranteed or endorsed by the publisher.

## References

[1] Chan J Brin MF Fahn S. Idiopathic cervical dystonia: clinical characteristics. Move Disord. (1991) 6:119–26. 10.1002/mds.870060206 2057004

[2] Jinnah HA Berardelli A Comella C Defazio G Delong MR Factor S. The focal dystonias: current views and challenges for future research. Move Disord. (2013) 28:926–43. 10.1002/mds.25567 23893450 PMC3733486

[3] Jankovic J Leder S Warner D Schwartz K. Cervical dystonia: clinical findings and associated movement disorders. Neurology. (1991) 41:1088–91. 10.1212/WNL.41.7.1088 2067638

[4] Brashear A. Botulinum toxin type A in the treatment of patients with cervical dystonia. Biologics. (2009) 3:1–7. 19707390 PMC2726049

[5] Charles PD Adams AM Davis T Bradley K Schwartz M Brin MF. Neck pain and cervical dystonia: treatment outcomes from cd probe (cervical dystonia patient registry for observation of onabotulinumtoxina efficacy). Pain Pract. (2016) 16:1073–82. 10.1111/papr.12408 26910788

[6] Martikainen KK Luukkaala TH Marttila RJ. Working capacity and cervical dystonia. Parkinsonism Relat Disord. (2010) 16:215–7. 10.1016/j.parkreldis.2009.07.006 19660976

[7] Richardson SP. Enhanced dorsal premotor-motor inhibition in cervical dystonia. Clin Neurophysiol. (2015) 126:1387–91. 10.1016/j.clinph.2014.10.140 25468241 PMC4409916

[8] Molho ES Stacy M Gillard P Charles D Adler CH Jankovic J. Impact of cervical dystonia on work productivity: an analysis from a patient registry. Mov Disord Clin Pract. (2016) 3:130–8. 10.1002/mdc3.12238 27774495 PMC5064605

[9] Berardelli A Rothwell JC Hallett M Thompson PD Manfredi M Marsden CD. The pathophysiology of primary dystonia. Brain. (1998) 121(Pt 7):1195–212. 10.1093/brain/121.7.1195 9679773

[10] Hallett M. Neurophysiology of dystonia: the role of inhibition. Neurobiol Dis. (2011) 42:177–84. 10.1016/j.nbd.2010.08.025 20817092 PMC3016461

[11] Hendrix CM Vitek JL. Toward a network model of dystonia. Ann N Y Acad Sci. (2012) 1265:46–55. 10.1111/j.1749-6632.2012.06692.x 22823747

[12] Moll CK Galindo-Leon E Sharott A Gulberti A Buhmann C Koeppen JA. Asymmetric pallidal neuronal activity in patients with cervical dystonia. Front Syst Neurosci. (2014) 8:15. 10.3389/fnsys.2014.00015 24574981 PMC3920073

[13] Draganski B Thun-Hohenstein C Bogdahn U Winkler J May A. “Motor circuit” gray matter changes in idiopathic cervical dystonia. Neurology. (2003) 61:1228–31. 10.1212/01.WNL.0000094240.93745.83 14610125

[14] Prell T Peschel T Kohler B Bokemeyer MH Dengler R Gunther A. Structural brain abnormalities in cervical dystonia. BMC Neurosci. (2013) 14:123. 10.1186/1471-2202-14-123 24131497 PMC3852757

[15] Obermann M Yaldizli O de Greiff A Lachenmayer ML Buhl AR Tumczak F. Morphometric changes of sensorimotor structures in focal dystonia. Mov Disord. (2007) 22:1117–23. 10.1002/mds.21495 17443700

[16] Zoons E Booij J Nederveen AJ Dijk JM Tijssen MA. Structural, functional and molecular imaging of the brain in primary focal dystonia–a review. Neuroimage. (2011) 56:1011–20. 10.1016/j.neuroimage.2011.02.045 21349339

[17] Egger K Mueller J Schocke M Brenneis C Rinnerthaler M Seppi K. Voxel based morphometry reveals specific gray matter changes in primary dystonia. Mov Disord. (2007) 22:1538–42. 10.1002/mds.21619 17588241

[18] Ramdhani RA Simonyan K. Primary dystonia: conceptualizing the disorder through a structural brain imaging lens. Tremor Other Hyperkinet Mov. (2013) 3:tre-03-152-3638-4. 10.7916/D8H70DJ7 23610744 PMC3629863

[19] Fabbrini G Pantano P Totaro P Calistri V Colosimo C Carmellini M. Diffusion tensor imaging in patients with primary cervical dystonia and in patients with blepharospasm. Eur J Neurol. (2008) 15:185–9. 10.1111/j.1468-1331.2007.02034.x 18217887

[20] Waugh JL Kuster JK Levenstein JM Makris N Multhaupt-Buell TJ Sudarsky LR. Thalamic volume is reduced in cervical and laryngeal dystonias. PLoS ONE. (2016) 11:e0155302. 10.1371/journal.pone.0155302 27171035 PMC4865047

[21] Colosimo C Pantano P Calistri V Totaro P Fabbrini G Berardelli A. Diffusion tensor imaging in primary cervical dystonia. J Neurol Neurosurg Psychiatry. (2005) 76:1591–3. 10.1136/jnnp.2004.056614 16227560 PMC1739383

[22] Delnooz CC Pasman JW Beckmann CF van de Warrenburg BP. Altered striatal and pallidal connectivity in cervical dystonia. Brain Struct Funct. (2015) 220:513–23. 10.1007/s00429-013-0671-y 24259114

[23] Delnooz CC Pasman JW Beckmann CF van de Warrenburg BP. Task-free functional MRI in cervical dystonia reveals multi-network changes that partially normalize with botulinum toxin. PLoS ONE. (2013) 8:e62877. 10.1371/journal.pone.0062877 23650536 PMC3641096

[24] Wei S Lu C Chen X Yang L Wei J Jiang W. Abnormal regional homogeneity and its relationship with symptom severity in cervical dystonia: a rest state fMRI study. BMC Neurol. (2021) 21:55. 10.1186/s12883-021-02079-x 33546628 PMC7863325

[25] Uddin LQ Kelly AM Biswal BB Margulies DS Shehzad Z Shaw D. Network homogeneity reveals decreased integrity of default-mode network in ADHD. J Neurosci Methods. (2008) 169:249–54. 10.1016/j.jneumeth.2007.11.031 18190970

[26] Wei S Su Q Jiang M Liu F Yao D Dai Y. Abnormal default-mode network homogeneity and its correlations with personality in drug-naive somatization disorder at rest. J Affect Disord. (2016) 193:81–8. 10.1016/j.jad.2015.12.052 26771948

[27] Guo W Yao D Jiang J Su Q Zhang Z Zhang J. Abnormal default-mode network homogeneity in first-episode, drug-naive schizophrenia at rest. Prog Neuropsychopharmacol Biol Psychiatry. (2014) 49:16–20. 10.1016/j.pnpbp.2013.10.021 24216538

[28] Guo W Liu F Zhang J Zhang Z Yu L Liu J. Abnormal default-mode network homogeneity in first-episode, drug-naive major depressive disorder. PLoS ONE. (2014) 9:e91102. 10.1371/journal.pone.0091102 24609111 PMC3946684

[29] Guo W Liu F Yao D Jiang J Su Q Zhang Z. Decreased default-mode network homogeneity in unaffected siblings of schizophrenia patients at rest. Psychiatry Res. (2014) 224:218–24. 10.1016/j.pscychresns.2014.08.014 25242670

[30] Pan P Wei S Ou Y Jiang W Li W Lei Y. Reduced global-brain functional connectivity and its relationship with symptomatic severity in cervical dystonia. Front Neurol. (2020) 10:1358. 10.3389/fneur.2019.01358 31998218 PMC6965314

[31] Jiang W Lei Y Wei J Yang L Wei S Yin Q. Alterations of interhemispheric functional connectivity and degree centrality in cervical dystonia: a resting-state fMRI study. Neural Plast. (2019) 2019:7349894. 10.1155/2019/7349894 31178903 PMC6507243

[32] Tsui JK Eisen A Stoessl AJ Calne S Calne DB. Double-blind study of botulinum toxin in spasmodic torticollis. Lancet. (1986) 2:245–7. 10.1016/S0140-6736(86)92070-2 2874278

[33] Matlab (2012). Available online at: www.mathworks.com

[34] Chao-Gan Y Yu-Feng Z. DPARSF: A MATLAB toolbox for “pipeline” data analysis of resting-state fMRI. Front Syst Neurosci. (2010) 4:13. 10.3389/fnsys.2010.00013 20577591 PMC2889691

[35] Kurata K. Somatotopy in the human supplementary motor area. Trends Neurosci. (1992) 15:159–60. 10.1016/0166-2236(92)90164-4 1377420

[36] Santosh CG Rimmington JE Best JJ. Functional magnetic resonance imaging at 1 T: motor cortex, supplementary motor area and visual cortex activation. Br J Radiol. (1995) 68:369–74. 10.1259/0007-1285-68-808-369 7795972

[37] Ramos VF Karp BI Hallett M. Tricks in dystonia: ordering the complexity. J Neurol Neurosurg Psychiatry. (2014) 85:987–93. 10.1136/jnnp-2013-306971 24487380 PMC4747630

[38] Naumann M Magyar-Lehmann S Reiners K Erbguth F Leenders KL. Sensory tricks in cervical dystonia: perceptual dysbalance of parietal cortex modulates frontal motor programming. Ann Neurol. (2000) 47:322−8. 10716251

[39] Franco AR Pritchard A Calhoun VD Mayer AR. Interrater and intermethod reliability of default mode network selection. Hum Brain Mapp. (2009) 30:2293–303. 10.1002/hbm.20668 19206103 PMC2751639

[40] Li W Qin W Liu H Fan L Wang J Jiang T. Subregions of the human superior frontal gyrus and their connections. Neuroimage. (2013) 78:46–58. 10.1016/j.neuroimage.2013.04.011 23587692

[41] Gusnard DA Akbudak E Shulman GL Raichle ME. Medial prefrontal cortex and self-referential mental activity: relation to a default mode of brain function. Proc Natl Acad Sci U S A. (2001) 98:4259–64. 10.1073/pnas.071043098 11259662 PMC31213

[42] Vogt BA Finch DM Olson CR. Functional heterogeneity in cingulate cortex: the anterior executive and posterior evaluative regions. Cereb Cortex. (1992) 2:435–43. 10.1093/cercor/2.6.435-a 1477524

[43] Mohan A Roberto AJ Mohan A Lorenzo A Jones K Carney MJ. The significance of the default mode network (DMN) in neurological and neuropsychiatric disorders: a review. Yale J Biol Med. (2016) 89:49–57. 27505016 PMC4797836

[44] Guo W Xiao C Liu G Wooderson SC Zhang Z Zhang J. Decreased resting-state interhemispheric coordination in first-episode, drug-naive paranoid schizophrenia. Prog Neuropsychopharmacol Biol Psychiatry. (2014) 48:14–9. 10.1016/j.pnpbp.2013.09.012 24075897

[45] de Vries PM Johnson KA de Jong BM Gieteling EW Bohning DE George MS. Changed patterns of cerebral activation related to clinically normal hand movement in cervical dystonia. Clin Neurol Neurosurg. (2008) 110:120–8. 10.1016/j.clineuro.2007.09.020 18006221

[46] Haaland KY Prestopnik JL Knight RT Lee RR. Hemispheric asymmetries for kinematic and positional aspects of reaching. Brain. (2004) 127(Pt 5):1145–58. 10.1093/brain/awh133 15033898

